# Thrombin-activatable fibrinolysis inhibitor and organ dysfunction in disseminated intravascular coagulation associated with sepsis

**DOI:** 10.1186/cc10370

**Published:** 2011-10-27

**Authors:** M Hayakawa, A Sawamura, M Sugano, S Uegaki, N Kubota, S Gando, S Jesmin

**Affiliations:** 1 Department of Anesthesiology and Critical Care Medicine, Hokkaido University Graduate School of Medicine, Sapporo, Japan; 2 Division of Gene Therapeutics, National Center for Global Health and Medicine, Tokyo, Japan

## Introduction

Fibrinolytic shutdown plays a pivotalrole in the pathogenesis of multiple organ dysfunction syndrome (MODS) in disseminated intravascular coagulation (DIC). We tested the hypothesis that the levels of thrombin activatable fibrinolysis inhibitor (TAFI) are not sufficient to overcome fibrinolytic shutdown, thus contributing to MODS and the poor prognosis in sepsis-induced DIC.

## Methods

Fifty patients with sepsis, severe sepsis, or septic shock were enrolled in the study. The DIC was diagnosed based on the Japanese Association for Acute Medicine (JAAM) DIC criteria. The overt DIC scores based on the International Society on Thrombosis and Haemostasis (ISTH) were also calculated. On the day of sepsis diagnosis (day 1), and days 3 and 5, we measured TAFI, soluble fibrin, and global coagulation and fibrinolysis markers.

## Results

The JAAM DIC scores on day 1 and maximum JAAM DIC scores were independent predictors of patient death and MODS, respectively. The JAAM DIC patients, especially those who simultaneously met the ISTH overt DIC criteria, showed lower TAFI antigen levels and activity, and higher levels of soluble fibrin in comparison with non-DIC patients. There were differences in the levels of soluble fibrin and TAFI activity between the patients with and without MODS. The findings of stepwise logistic regression and multiple regression analyses suggested that low TAFI activity is an independent predictor of patient death and MODS. A multiple regression analysis also indicated that soluble fibrin negatively correlated with the TAFI activity in DIC patients.

## Conclusion

Thrombin activation results in the consumption of TAFI. Low TAFI activity is involved in the pathogenesis of DIC-induced MODS and poor prognosis.

